# Ultra-processed food consumption and obesity in the Australian adult population

**DOI:** 10.1038/s41387-020-00141-0

**Published:** 2020-12-05

**Authors:** Priscila Pereira Machado, Eurídice Martinez Steele, Renata Bertazzi Levy, Maria Laura da Costa Louzada, Anna Rangan, Julie Woods, Timothy Gill, Gyorgy Scrinis, Carlos Augusto Monteiro

**Affiliations:** 1 Institute for Physical Activity and Nutrition, School of Exercise and Nutrition Sciences, Deakin University, Geelong, VIC 3220 Australia; 2 Center for Epidemiological Research in Nutrition and Health, University of Sao Paulo, Av. Dr. Arnaldo, 715, Sao Paulo, 01246-904 Brazil; 3 Departamento de Medicina Preventiva, Faculdade de Medicina, Universidade de São Paulo, Av. Dr. Arnaldo, 455, Sao Paulo, 01246-903 Brazil; 4 Departamento de Nutrição, Faculdade de Saúde Pública, Universidade de São Paulo, Av. Dr. Arnaldo, 715, Sao Paulo, 01246-904 Brazil; 5 School of Life and Environmental Sciences, Charles Perkins Centre, The University of Sydney, Camperdown, NSW 2050 Australia; 6 Boden Institute of Obesity, Nutrition, Exercise and Eating Disorders, Charles Perkins Centre, The University of Sydney, Camperdown, NSW 2050 Australia; 7 School of Agriculture and Food, The University of Melbourne, Parkville, Melbourne, VIC 3010 Australia

**Keywords:** Obesity, Risk factors

## Abstract

**Background:**

Rapid simultaneous increases in ultra-processed food sales and obesity prevalence have been observed worldwide, including in Australia. Consumption of ultra-processed foods by the Australian population was previously shown to be systematically associated with increased risk of intakes of nutrients outside levels recommended for the prevention of obesity. This study aims to explore the association between ultra-processed food consumption and obesity among the Australian adult population and stratifying by age group, sex and physical activity level.

**Methods:**

A cross-sectional analysis of anthropometric and dietary data from 7411 Australians aged ≥20 years from the National Nutrition and Physical Activity Survey 2011–2012 was performed. Food consumption was evaluated through 24-h recall. The NOVA system was used to identify ultra-processed foods, i.e. industrial formulations manufactured from substances derived from foods and typically added of flavours, colours and other cosmetic additives, such as soft drinks, confectionery, sweet or savoury packaged snacks, microwaveable frozen meals and fast food dishes. Measured weight, height and waist circumference (WC) data were used to calculate the body mass index (BMI) and diagnosis of obesity and abdominal obesity. Regression models were used to evaluate the association of dietary share of ultra-processed foods (quintiles) and obesity indicators, adjusting for socio-demographic variables, physical activity and smoking.

**Results:**

Significant (*P*-trend ≤ 0.001) direct dose–response associations between the dietary share of ultra-processed foods and indicators of obesity were found after adjustment. In the multivariable regression analysis, those in the highest quintile of ultra-processed food consumption had significantly higher BMI (0.97 kg/m^2^; 95% CI 0.42, 1.51) and WC (1.92 cm; 95% CI 0.57, 3.27) and higher odds of having obesity (OR = 1.61; 95% CI 1.27, 2.04) and abdominal obesity (OR = 1.38; 95% CI 1.10, 1.72) compared with those in the lowest quintile of consumption. Subgroup analyses showed that the trend towards positive associations for all obesity indicators remained in all age groups, sex and physical activity level.

**Conclusion:**

The findings add to the growing evidence that ultra-processed food consumption is associated with obesity and support the potential role of ultra-processed foods in contributing to obesity in Australia.

## Introduction

Non-communicable diseases (NCDs) are estimated to account for 89% of all deaths in Australia^1^, and high body mass index (BMI) remains the second greatest risk factor driving most death and disability in the country^1^. In the past 20 years, Australian prevalence of obesity has risen dramatically—19% in 1995, 27% in 2012 and 31% in 2018^2^—and currently has the fifth highest rate of obesity among the Organisation for Economic Co-operation and Development countries^3^.

The increase in global obesity rates appears to be a consequence of changes in global food systems^4,5^, leading to the displacement of dietary patterns based on traditional meals by those that are increasingly made up of ultra-processed foods^6^. A growing body of evidence from cross-sectional and longitudinal studies conducted worldwide has shown that ultra-processed food consumption is consistently associated with weight gain and obesity^7–14^.

Ultra-processed foods are defined by the NOVA food classification system as industrial formulations manufactured from substances derived from foods (e.g. modified starch, maltodextrin, hydrogenated oils, protein isolates) and typically added of flavours, colours and other cosmetic additives^15^. The poor nutrient profile of these foods (high in salt or added sugar and unhealthy fats and low in dietary fibre, micronutrients and phytochemicals) and the processing itself (altered physical and structural characteristics, removal of water and use of flavours, flavour enhancers, colours and other cosmetic additives) make them intrinsically nutritionally unbalanced, hyper-palatable and habit-forming. They dispense the necessity of culinary preparation and are ubiquitous, which make them convenient and accessible. Their manufacture using low-cost ingredients and the aggressive marketing of these products amplify their market advantages over unprocessed or minimally processed foods and freshly prepared meals. All those factors contribute to the replacement of traditional dietary patterns by others based on ultra-processed foods and also encourage the excessive consumption of energy^15,16^, conditions potentially related to the increased risk of obesity^16^.

A recent randomised controlled trial (RCT) showed that, compared to a diet with no ultra-processed foods, a diet with >80% of ultra-processed foods caused an increase in energy intake of near 500 kcal per day and that, in 2 weeks, participants exposed to the ultra-processed diet gained 0.9 kg while participants exposed to the non-ultra-processed diet lost 0.9 kg^17^. Interestingly, this was despite both diets being designed to be matched for energy, macronutrients, fibre and sodium content, suggesting that mechanisms other than nutrient profile might explain observed differences.

Ultra-processed food sales are increasing globally, including in Australia^13,18^. A previous study based on the Australian 2011–2012 National Nutrition and Physical Activity survey found that the increased dietary share of ultra-processed foods was systematically associated with intakes of nutrients outside levels recommended for the prevention of obesity and other NCDs^19^. This same survey simultaneously collected food intake and anthropometric data and thus allows testing of the hypothesis that increases in the dietary share of ultra-processed foods are associated with increases in the risk of obesity among the adult population, regardless of age group, sex and physical activity level.

## Methods

### Data source and collection

The data source is the National Nutrition and Physical Activity Survey (NNPAS), a household survey that collects information about the Australian population’s health, including anthropometric, food consumption and physical activity data. This survey recruited a random sample of the Australian population obtained by using a complex, stratified, multistage probability cluster sampling design based on the selection of strata, households and people within households. The NNPAS was conducted between May 2011 and June 2012, covering 9519 households where 12,153 Australians were interviewed. Information was obtained about one adult and, where possible, one child aged 2–17 years in each selected household^20^.

### Food consumption

Data on food consumption were collected based on two non-consecutive 24-h dietary recalls using an electronic survey, which guides the interviewer towards preventing the interviewee from forgetting consumption items frequently omitted by those interviewed (USDA Automated Multiple-Pass Method). The first recall was applied through a face-to-face interview (*n* = 12,153) while the second recall (*n* = 7735) was applied via a telephone interview conducted ≥8 days after the first interview^20^.

Energy was estimated based on the Australian Food and Nutrient Database (AUSNUT 2011–2013), which contains information for 5740 foods and beverages consumed during the survey^21^.

Mixed dishes composed of two or more food items were disaggregated using the AUSNUT 2011–2013 Food Recipe File (45% of food codes). Reported single food items and the underlying ingredients of mixed dishes were classified according to the NOVA classification system into the following four groups (and subgroups within these groups): (a) unprocessed or minimally processed foods, e.g. cereals, legumes, vegetables, fruits, milk, meat; (b) processed culinary ingredients, e.g. table salt, table sugar, honey, vegetable oils, butter; (c) processed foods, e.g. canned vegetables in brine, salted or sugared nuts, canned fish, freshly made breads and cheeses; and (d) ultra-processed foods, e.g. mass-produced packaged breads, carbonated soft drinks, confectionery, cookies, breakfast ‘cereals’, flavoured yoghurts, reconstituted meat products, ready to heat meals and packaged instant soups and noodles^15^. More information regarding the application of the NOVA system to AUSNUT 2011–13 and access to the coding can be found elsewhere(20).

### Obesity indicators

Weight, height and waist circumference (WC) measurements were obtained and registered in the surveys by interviewers using digital scales, vertical stadiometers and a metal tape measure, respectively, following standard measurement techniques^20^. BMI (weight (kg)/height (m)^2^) and WC (cm) were used as indicators of adiposity. Obesity was defined as BMI ≥ 30 kg/m^2^ ^22^, and abdominal obesity as WC ≥88 cm for women and ≥102 cm for men^23^.

### Covariates

Demographic covariates of interest include age, sex, educational attainment, socio-economic status (assessed with the Socio-Economic Index of Disadvantage for Areas (SEIFA), a ranking based on the relative socio-economic advantage and disadvantage of the location of the household), zones (urbanity of the household location based on the Australian Standard Geographical Classification) and country of birth. Physical activity, based on total minutes undertaken in physical activity for fitness, recreation, sport or (for) transport in last week, and current smoking status were also accounted in the analysis^20^.

### Inclusion and exclusion criteria

**Fig. 1:**
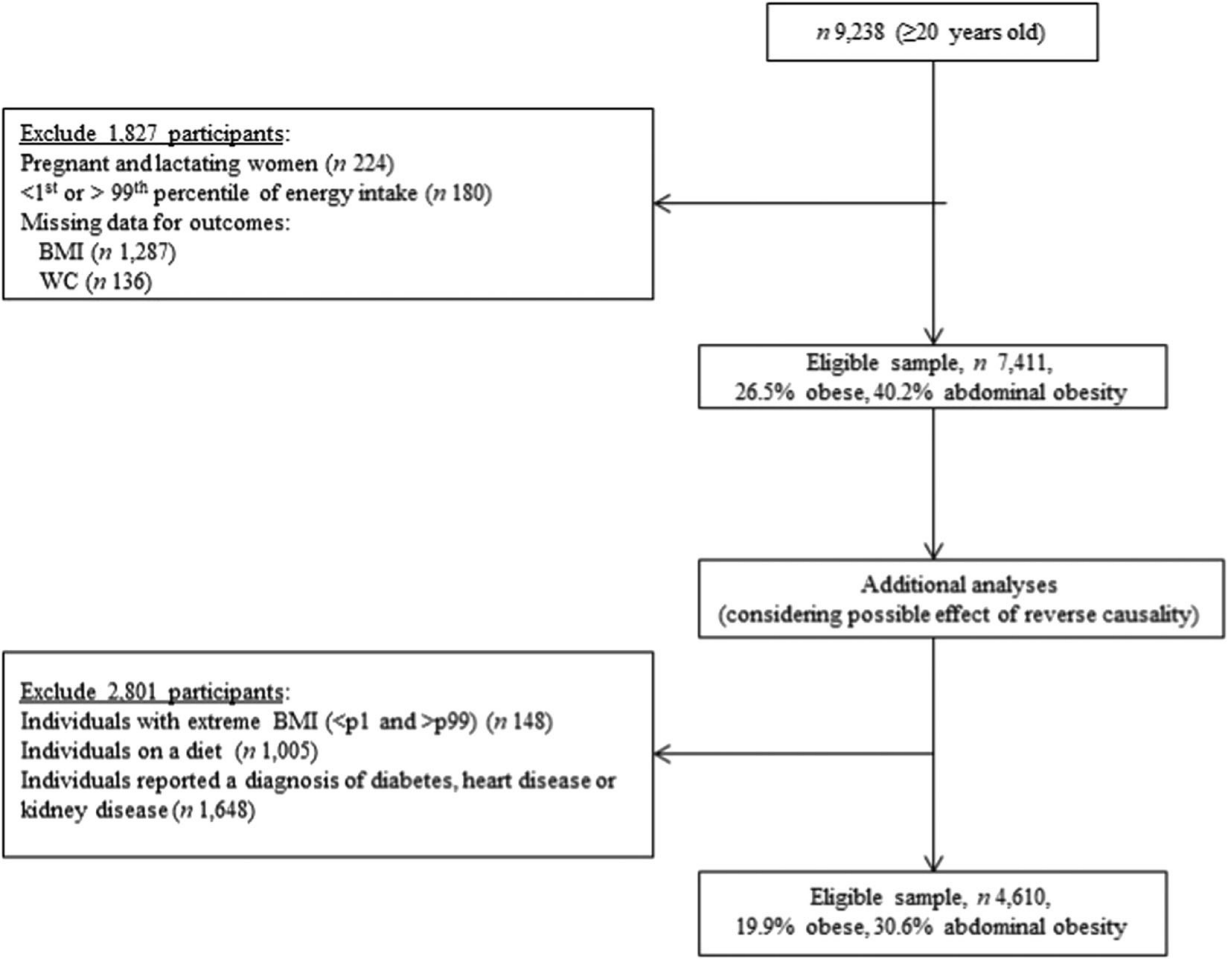
Flowchart showing participants excluded in each analysis (NNPAS 2011–2012). Eligibility criteria of study participants. Number of people excluded presented between parentheses.

The analytical sample was restricted to adults aged 20–85 years. Individuals were included in the analyses if they had complete data for BMI and WC. A total of 9238 participants were in the appropriate age span and eligible to be included in the analyses. Of these, pregnant and lactating women, participants who reported implausible energy intakes (<1st or >99th percentile of energy intake) and individuals with missing data for outcomes were excluded. All individuals presented information for the exposure. Thus the final sample of this study was 7411 (Fig. 1).

### Data analysis

The first 24-h recall was used for the analyses. The population was first stratified into quintiles of the dietary share of ultra-processed foods (percentage of total energy intake), with the lowest consumers belonging to the first quintile and the highest consumers to the fifth. Thereafter, the characteristics of participants (demographics, physical activity, smoking status and total energy intake) according to quintiles of ultra-processed food consumption were assessed. Differences in those characteristics across the dietary share of ultra-processed foods were evaluated by Pearson’s *χ*^2^ test of independence (categorical variables) and unadjusted linear regression models (treating quintile of ultra-processed food consumption as an ordinal variable).

Linear and logistic regression analyses were performed to assess the association between the dietary contribution of ultra-processed foods (quintiles) and obesity indicators, i.e. BMI (as a continuous variable and categorised to identify obesity) and WC (continuous and categorised to identify abdominal obesity). For all outcomes, we ran an unadjusted model and thereafter a multivariable model adjusted for sociodemographic variables, physical activity and smoking status. Multivariable adjusted subgroups analysis using ultra-processed food consumption as continuous were performed for age group (20–39, 40–59, ≥60 years), sex (male, female) and physical activity level (active, inactive).

Additional multivariable adjusted analysis to account for potential effect of reverse causality in the relationship between ultra-processed food consumption (quintiles) and obesity indicators were performed excluding 2 801 individuals with extreme BMI values, following ‘special diets’ (i.e. on a diet to lose weight and/or for health reasons) at the time of the survey, or who reported a diagnosis of diabetes, heart disease or kidney disease, which could be associated with long term dietary behaviour change (Fig. 1).

All multivariable regression models were adjusted for sex (male/female), age (continuous), years of education (completed ≤9 years including never attended, completed 10–12 years with no graduate degree, completed 12 years with graduate degree), income (SEIFA—quintiles), zones (major cities of Australia, inner regional and other, which includes outer regional, remote and very remote Australia), country of birth (Australia or English country/other), level of physical activity (inactive/active, classified as active when physical activity last week met the 150 min recommended guidelines) and smoking status (never smoked, former smoker and current smoker). The fit of the model was verified by residual distribution plots, which should follow a normal distribution.

Weighted analyses were performed using Stata survey module (version 14) to consider the effect of complex sampling procedures adopted in the NNPAS 2011–2012 and in order to allow extrapolation of results for the Australian population (Stata Corp., College Station, United States).

This study was a secondary analysis using de-identified data from the ABS Basic Confidentialised Unit Record Files, and permission to use the data was obtained. Ethics approval for the survey was granted by the Australian Government Department of Health and Ageing Departmental Ethnics Committee in 2011^20^.

## Results

Table 1 describes characteristics of the overall Australian adult population and of strata of this population that correspond to quintiles of the dietary share of ultra-processed foods. Ultra-processed foods represented 38.9% of total energy intake among Australian adults, ranging from 12.7% (range 0–21.7%) in the lowest quintile of ultra-processed food consumption to 74.2% (range 62.1–100%) in the highest quintile. Compared with participants in the lowest quintile, individuals in the highest quintile of ultra-processed food consumption were younger (Q5 = 40.8 vs. Q1 = 48.4 years, *P* < 0.001), more likely to belong to the poorest SEIFA quintile (22.8 vs. 15.5%, *P* < 0.001), be Australian or from English country (88.0 vs. 70.3%, *P* < 0.001), inactive (53.5 vs. 41.8%, *P* < 0.001), current smoker (26.4 vs. 17.0%, *P* < 0.001) and have higher total energy intake (8 951.8 vs. 8 055.9 kJ, *P* < 0.001) and less likely to be higher educated (17.7 vs. 29.9%, *P* < 0.001) and to live in major cities (68.6 vs. 75.2%, *P* = 0.002) (Table 1).

**Table 1:** Characteristics of the population according to dietary share of ultra-processed foods.

	Dietary share of ultra-processed foods (quintiles)^a^						
	All	Q1	Q2	Q3	Q4	Q5	*P* value*
*Age group (%)*							<0.001
20–39 years	38.5	31.8	22.7	25.5	40.9	56.3	
40–59 years	36.4	41.7	38.7	38.0	32.3	28.0	
≥60 years	25.1	26.5	27.6	26.5	26.8	15.7	
*Sex (%)*							0.493
Male	51.7	50.4	50.7	51.7	53.5	52.9	
Female	48.3	49.6	49.3	48.3	46.5	47.1	
*Years of education (%)*							<0.001
≤9 years	13.5	13.7	13.3	13.8	14.4	12.1	
10–12 years	61.8	56.4	59.3	62.4	64.1	70.2	
10–12 years with graduate degree	24.7	29.9	27.4	23.8	21.5	17.7	
*SEIFA (%)*							<0.001
Quintile 1—greater disadvantage	17.7	15.5	16.2	16.6	19.6	22.8	
Quintile 2	19.9	18.7	20.4	19.9	19.9	20.7	
Quintile 3	21.1	19.7	18.9	23.4	23.0	21.4	
Quintile 4	19.1	19.8	21.6	18.8	17.3	16.3	
Quintile 5—greater advantage	22.2	26.3	22.8	21.4	20.2	18.8	
*Zones (%)*							0.002
Major cities	71.5	75.2	72.9	72.9	66.4	68.6	
Inner regional	19.3	16.5	18.6	18.0	22.5	22.5	
Other	9.2	8.3	8.5	9.1	11.1	8.9	
*Country of birth (%)*							<0.001
Australia or English country	79.8	70.3	77.1	81.7	86.4	88.0	
Other	20.2	30.0	22.8	18.2	13.5	12.0	
*Physical activity level (%)*^b^							<0.001
Inactive	48.0	41.8	47.1	47.5	53.1	53.5	
Active	52.0	58.2	52.9	52.5	46.9	46.5	
*Smoking status (%)*							<0.001
Never smoked	49.8	50.4	52.1	51.3	48.2	45.7	
Former smoker	31.8	32.6	33.4	31.8	32.2	27.9	
Current smoker	18.4	17.0	14.5	16.9	19.6	26.4	
*Total energy intake (kJ)*^c^	8421.9	8055.9	8388.7	8376.2	8523.1	8951.8	<0.001

Australian population aged ≥20 years (NNPAS 2011–2012), *n* = 7411.

*P value for continuous variables is estimated through unadjusted linear regression, treating quintile of ultra-processed food consumption as an ordinal variable, and Pearson’s *χ*^2^ for categorical variables.

^a^Percentage of energy intake from ultra-processed foods. Mean (range): All = 38.9 (0–100); Q1 = 12.7 (0–21.7); Q2 = 28.4 (21.7–34.6); Q3 = 40.3 (34.6–46.6); Q4 = 54.0 (46.6–62.1); Q5 = 74.2 (62.1–100).

^b^Active whether physical activity last week met 150 min recommended guidelines.

^c^1 kcal = 4.186 kJ.

The mean BMI and WC in the Australian adult population were 27.4 kg/m^2^ and 92.8 cm, respectively (Table 2), whereas the prevalence of obesity and abdominal obesity was 26.5% and 40.2%, respectively (Table 3). Crude and multivariable models showed that the dietary share of ultra-processed foods was significantly associated with higher BMI and WC (Table 2) and greater prevalence of both obesity and abdominal obesity among Australian adults (Table 3) (*P*-trend ≤ 0.001 for all outcomes). Significant direct dose–response associations between the dietary share of ultra-processed foods and BMI (Table 2) and obesity (Table 3) were found after adjusting for sociodemographic variables, physical activity and smoking. In the multivariable regression analyses, we observed that those in the highest quintile of ultra-processed food consumption had mean BMI 0.97 kg/m^2^ (95% confidence interval (CI) 0.42; 1.51) and WC 1.92 cm (95% CI 0.57; 3.27) higher compared with those in the lowest quintile of consumption (Table 2). The adjusted odds ratios (ORs) of having obesity and abdominal obesity were, respectively, 1.61 (95% CI 1.27; 2.04) and 1.38 (95% CI 1.10; 1.72) in the top quintile of ultra-processed food consumption in regard to the lowest (Table 3).

**Table 2:** Association of dietary share of ultra-processed foods (% of total energy) with BMI and WC among Australians aged ≥20 years (NNPAS 2011–2012), *n* = 7411.

Quintiles of the dietary contribution of ultra-processed foods (% of total dietary energy)^a^	BMI (kg/m^2^)					WC (cm)				
	Mean	Mean difference	(95% CI)	Mean difference, adjusted^b^	(95% CI)	Mean	Mean difference	(95% CI)	Mean difference, adjusted^b^	(95% CI)
Q1 (lowest)	26.7	0.00	Ref.	0.00	Ref.	91.1	0.00	Ref.	0.00	Ref.
Q2	27.3	0.66	(0.21; 1.12)	0.52	(0.07; 0.95)	92.9	1.73	(0.48; 2.97)	1.26	(0.19; 2.33)
Q3	27.6	0.86	(0.38; 1.32)	0.66	(0.20; 1.11)	93.2	2.00	(0.71; 3.29)	1.42	(0.30; 2.54)
Q4	27.9	1.26	(0.77; 1.75)	0.96	(0.47; 1.45)	94.8	3.63	(2.31; 4.94)	2.66	(1.46; 3.87)
Q5 (highest)	27.7	1.06*	(0.50; 1.61)	0.97*	(0.42; 1.51)	92.9	1.77*	(0.29; 3.26)	1.92*	(0.57; 3.27)
Total	27.4	–	–	–	–	92.8	–	–	–	–

*BMI* body mass index, *WC* waist circumference, *CI* confidence interval, *Ref*. reference group.

*P-trend ≤ 0.001.

^a^See previous table.

^b^Adjusted for sex, age, educational attainment, income, zones, country of birth, level of physical activity and smoking status.

**Table 3:** Association of dietary share of ultra-processed foods (% of total energy) with obesity and abdominal obesity among Australians aged ≥20 years (NNPAS 2011–2012), *n* = 7411.

Quintiles of the dietary contribution of ultra-processed foods (% of total dietary energy)^a^	Obesity (BMI ≥ 30 kg/m^2^)					Abdominal obesity^b^				
	Percent	OR	(95% CI)	OR, adjusted^c^	(95% CI)	Percent	OR	(95% CI)	OR, adjusted^c^	(95% CI)
Q1 (lowest)	20.7	1.00	Ref.	1.00	Ref.	35.1	1.00	Ref.	1.00	Ref.
Q2	26.3	1.36	(1.11; 1.67)	1.29	(1.05; 1.59)	41.0	1.29	(1.07; 1.55)	1.24	(1.02; 1.51)
Q3	27.2	1.43	(1.16; 1.76)	1.33	(1.07; 1.64)	39.7	1.22	(1.01; 1.47)	1.16	(0.95; 1.42)
Q4	29.9	1.62	(1.30; 2.00)	1.44	(1.15; 1.80)	46.2	1.59	(1.31; 1.92)	1.53	(1.24; 1.88)
Q5 (highest)	30.9	1.71*	(1.36; 2.14)	1.61*	(1.27; 2.04)	40.5	1.26*	(1.03; 1.55)	1.38*	(1.10; 1.72)
Total	26.5	–	–	–	–	40.2	–	–	–	–

*BMI* body mass index, *OR* odds ratio, *CI* confidence interval, *Ref*. reference group.

*P-trend ≤ 0.001.

^a^See previous table.

^b^Defined as waist circumference ≥88 cm for women and ≥102 cm for men.

^c^Adjusted for sex, age, educational attainment, income, zones, country of birth, level of physical activity and smoking status.

Positive associations for all obesity indicators were also observed in stratified analysis across all age groups (though did not reach statistical significance with WC and abdominal obesity among the youngest), sex and physical activity level (Table 4). The association of ultra-processed food consumption on BMI and WC was stronger among people aged ≥40 years, female and inactive. The association of ultra-processed food consumption on obesity was stronger among people aged ≥60 years, male and inactive and on abdominal obesity was stronger among people aged ≥40 years, male and inactive. However, these differences were not statistically significant (Table 4).

**Table 4:** Association of dietary share of ultra-processed foods^a^ with indicators of adiposity by age, sex and physical activity level.

	Percentage of energy intake from ultra-processed foods	BMI (kg/m^2^)			WC (cm)			Obesity (BMI ≥ 30 kg/m^2^)			Abdominal obesity^c^		
		Mean	Mean difference^b^	(95% CI)	Mean	Mean difference^b^	(95% CI)	Percent	OR^b^	(95% CI)	Percent	OR^b^	(95% CI)
*Age, years*													
20–39	43.4	26.1	0.15	(0.02 to 0.28)	88.2	0.27	(−0.06 to 0.60)	18.5	1.09	(1.02 to 1.16)	24.8	1.05	(0.99 to 1.11)
40–59	36.2	28.1	0.20	(0.08 to 0.32)	94.6	0.59	(0.29 to 0.89)	30.9	1.08	(1.02 to 1.14)	44.6	1.08	(1.03 to 1.14)
≥60	36.2	28.5	0.22	(0.05 to 0.39)	97.6	0.37	(0.02 to 0.77)	32.5	1.11	(1.04 to 1.18)	57.3	1.08	(1.01 to 1.14)
*Sex*													
Male	39.3	27.7	0.11	(0.02 to 0.21)	97.6	0.31	(0.05 to 0.57)	26.2	1.09	(1.03 to 1.14)	35.4	1.08	(1.03 to 1.13)
Female	38.5	27.1	0.23	(0.10 to 0.35)	87.8	0.47	(0.17 to 0.77)	26.9	1.07	(1.02 to 1.12)	45.2	1.05	(1.01 to 1.09)
*Physical activity level*													
Inactive	40.7	28.0	0.21	(0.08 to 0.32)	94.9	0.49	(0.20 to 0.78)	31.1	1.09	(1.04 to 1.14)	47.6	1.08	(1.03 to 1.13)
Active	37.3	26.9	0.14	(0.03 to 0.24)	91.0	0.29	(0.02 to 0.56)	22.3	1.07	(1.02 to 1.13)	33.3	1.05	(1.01 to 1.10)

Australian population aged ≥20 years (NNPAS 2011–2012), *n* = 7411.

*BMI* body mass index, *WC* waist circumference, *OR* odds ratio, *CI* confidence interval.

^a^For an increase of 10% of the proportion of ultra-processed food intake in the diet.

^b^Adjusted for sex, age, educational attainment, income, zones, country of birth, level of physical activity and smoking status.

^c^Defined as waist circumference ≥88 cm for women and ≥102 cm for men.

Additional analyses considering the potential effect of reverse causality showed an increase in the magnitude of the associations in the fifth quintile of ultra-processed food consumption (regarding the first) for all obesity indicators in comparison to the multivariable models performed in the full analytical sample. Significant direct dose–response associations between the dietary share of ultra-processed foods and WC and abdominal obesity were observed in these analyses (Table S1).

## Discussion

In this nationally representative cross-sectional study, the association of ultra-processed food consumption with obesity among Australian adults was investigated. It was found that higher consumption of ultra-processed foods was significantly associated with greater BMI and WC and greater odds of having obesity and abdominal obesity. Trend towards positive associations of ultra-processed food consumption and obesity indicators were observed in both men and women and across age groups (but not significantly associated among the youngest age groups) and levels of physical activity.

Australians whose diets were based on ultra-processed foods (>62% of total energy intake) had 0.97 units higher BMI, 1.92 cm greater WC and were 61 and 38% more likely of having obesity and abdominal obesity, respectively, than individuals whose diets were not based on ultra-processed foods (<22% of energy intake). The findings of the present study are supported by existing literature showing a causal relationship between ultra-processed food consumption and weight gain^17^. In an RCT conducted by Hall et al., at the end of 2 weeks participants gained, on average, 0.9 ± 0.3 kg during the ultra-processed diet and lost, on average, 0.9 ± 0.3 kg during the diet based on non-ultra-processed foods.

Also supporting our findings, consumption of ultra-processed foods was found to be associated with 9-year incidence of overweight or obesity in a prospective cohort of Spanish middle-aged adult university graduates^9^, incidence of obesity or of higher weight gain among Brazilian^14^ and U.K. adults^24^ and in cross-sectional studies involving nationally representative sample of adults in the U.S.^10^, Canada^12^ and Brazil^8^. An ecological study including 19 European countries found a significant positive association between national household availability of ultra-processed foods and national prevalence of obesity among adults^10^. Similarly, a study across 80 high- and middle-income countries found a positive association of annual changes in sales per capita of ultra-processed products with adult BMI trajectories^13^.

The mechanisms underlying the association between ultra-processed food consumption and obesity are not fully established and may result from a combination of the obesogenic nutritional profile of these foods^25^, non-nutritional mechanisms related to the processing itself^26–28^ and the displacement of nutritious unprocessed and minimally processed foods and fresh meals prepared with these foods^29^.

Population-based studies conducted in several countries have shown that the energy share of ultra-processed foods impacted negatively on the intake of nutrients linked to obesity, such as free or added sugars, total fats, dietary energy density and fibre^19,30–34^. In Australia, the risk of having diets that do not comply with dietary goals recommended for the prevention of obesity increased linearly across quintiles of dietary share of ultra-processed foods, attaining the astonishing 3.9 higher risk of excessive free sugar intake among the highest consumers of ultra-processed foods^19^.

Processing techniques applied in the manufacture of ultra-processed foods, such as the partial or total withdrawal of water, the deconstruction of the original food matrix structure and the use of high amounts of sugar, salt, fats, and cosmetic additives, which enhance oro-sensory properties and energy density of these foods, may increase eating rate (grams consumed per minute) and override endogenous satiety and appetite signalling, thereby resulting in greater overall intake^17,27,35,36^. Ultra-processed beverages may have an even stronger effect by adding to total energy intake without displacing energy from solid foods, as well as affecting subsequent meals due to incomplete compensatory reduction in energy intake^37^. Recent evidence indicates that part of this mechanism may be explained by alteration in the gut microbiota^26,38,39^. In fact, the role of food processing on the gut system has recently been emphasised with evolving evidence showing the effect of the gut microbiota on energy homoeostasis and lipid accumulation of the host, and consequent weight gain and obesity^26,39,40^. Besides the disruption of the gut–brain satiety signalling, diets based on ultra-processed foods may induce microbiota dysbiosis, gut inflammation and decrease gut barrier function due to the presence of non-caloric artificial sweeteners, emulsifiers, advanced glycation end products and the low content of micronutrients and phytosterols in these foods^26,38,39^.

Ultra-processed foods, such as soft drinks, ready meals, confectionaries and biscuits, are frequently designed to be convenient and able to be consumed anywhere, as snacks rather than as regular meals^6,16,29^. They are accessible^25^, affordable^41^, aggressively marketed^42–44^ and their portion sizes are increasing over time^45^. These characteristics may also contribute to the displacement of freshly prepared meals and stimulate overconsumption of energy^16^.

The consumption of ultra-processed foods is increasing worldwide, already comprising the majority of calories consumed in high-income countries, such as the U.K.^33^, the U.S.^32^ and Canada^31^. Besides weight gain and obesity, the consumption of ultra-processed foods has been associated with increased risk of all-cause mortality^46–48^, hypertension and cardiovascular diseases^49,50^, metabolic syndrome^51^, cancer^52^, diabetes^53^, depression^54^ and gastrointestinal disorders^55^.

Given the adverse outcomes related to ultra-processed food consumption, dietary advice and policy actions could be aimed at decreasing consumption of these foods, while promoting the availability, accessibility and affordability of unprocessed and minimally processed foods^16^. Furthermore, the role of food processing, in particular of ultra-processed foods in contributing to obesity in Australia, should be considered in the current discussion for an overarching strategy to tackle obesity in the country^56^.

This study has several strengths. To the best of our knowledge, this is the first study to analyse the association of ultra-processed food consumption with obesity in Australia. We used the most up-to-date, individual-level dietary survey data taken from a nationally representative sample of Australian adults, increasing generalisability. The analyses were based on the NOVA food classification system^15^, which has been recognised by UN agencies as a relevant approach for linking dietary intake, obesity and NCDs^16,57,58^. The NOVA system was applied into disaggregated food codes in the Australian food composition database, which enabled determining food processing level based on standardised, objective and clear criteria, reducing the chance of misclassification. The availability of sociodemographic, physical activity and smoking data allowed adjustment for several confounders, to test consistency among population groups and provided novel evidence on group-specific associations. Although BMI is considered a useful tool to assess body mass at the population level, the inclusion of a second indicator of adiposity (WC) strengthens our findings and adds valuable information on body fat distribution^59^.

Nevertheless, there are also limitations to the interpretation of the findings. First, this is a cross-sectional study, and thus temporality and causality cannot be established. However, the results are biologically plausible and consistent with the randomised controlled trial that has assessed the short-term impact of ultra-processed diets on energy intake and weight gain^17^, and with a few longitudinal studies that have assessed the association between the dietary share of ultra-processed foods and the incidence of obesity or of higher weight gain^9,14,24^. Besides, reverse causality cannot be ruled out. In fact, the magnitude of the associations increased in the sensitivity analyses excluding individuals on ‘special diets’, with extreme BMI values or with diagnosis of diet-related chronic diseases that may have changed dietary behaviour (Table S1). Although a vast array of potential confounders were controlled for, residual confounding due to unmeasured confounders (e.g. parity, menopause) could explain, at least in part, the observed associations.

In addition, limitations related to the dietary assessment instrument deserve mention. Analyses were based on a single recall and may not represent usual diet, possibly biasing studied association towards the null. Analyses were based on a single rather than two recalls because the lower response rate for the second day (64%) could have introduced sampling bias. Obesity could have changed individuals’ health behaviours, including diet, reducing overall intake of ultra-processed foods among obese people, hence attenuating the magnitude of the associations. Misreporting is an inherent potential bias of the 24-h recall. Some studies suggest that foods usually considered unhealthy (e.g. ultra-processed foods like confectionary, cakes, chips) are more likely to be under-reported^60^. However, this may be partly mitigated by having excluded participants who reported implausible energy intakes, and if differential information bias occurred, the associations would be biased towards the null. Finally, the dietary survey and food composition database were not designed specifically to categorise foods according to characteristics of industrial processing, and so some misclassification of foods at the individual level cannot be excluded. However, standardised, objective and clear criteria were considered, plus several independent researchers reviewed the classification, and a conservative approach (assigning lower level of processing) was used in case of uncertainty.

In conclusion, these findings add to the growing evidence that ultra-processed food consumption is associated with increased risk of obesity and support the potential role of ultra-processed foods in contributing to obesity in Australia. Despite the cross-sectional nature of the study, the results are biologically plausible and underpinned by evidence derived from experimental and cross-sectional and longitudinal studies from several high- and middle-income countries showing similar results. Importantly, this study contributes to the evolving evidence on the role of food processing in adiposity and is the first one to present an association between ultra-processed food consumption and obesity in a nationally representative sample of Australian adults.

Future studies should be extended to populations around the world, to present context-dependent magnitudes and drivers of ultra-processed food consumption and obesity. Mechanistic studies are needed to clarify underlying plausible causal pathways that explain links between food processing and adiposity. This evidence is relevant to inform policy makers and for dietary advice at the population and clinical levels.

## Supplementary information

Table S1. Association of dietary share of ultra-processed foodsa with indicators of adiposity considering potential effect of reverse causality†. Australians aged ≥20 years (NNPAS 2011–2012), n 4 610.
