# Supplementary material for: Ultra-processed food consumption and obesity in the Australian adult population
Source: Nutr Diabetes. 2020 Dec 5;10:39. doi: 10.1038/s41387-020-00141-0 (PMC7719194; doi:10.1038/s41387-020-00141-0)
Supplement: Supplementary file 1 — Table S1. Association of dietary share of ultra-processed foodsa with indicators of adiposity considering potential effect of reverse causality†. Australians aged ≥20 years (NNPAS 2011–2012), n 4 610. [file 41387_2020_141_MOESM1_ESM.docx]

**ULTRA-PROCESSED FOOD CONSUMPTION AND OBESITY IN THE AUSTRALIAN ADULT POPULATION**

**Supplementary Information**

| **Table S1.** Association of dietary share of ultra-processed foods^a^ with indicators of adiposity considering potential effect of reverse causality^†^. Australians aged ≥20 years (NNPAS 2011–2012), *n* 4 610. | | | | | | | | | | | | |
| --- | --- | --- | --- | --- | --- | --- | --- | --- | --- | --- | --- | --- |
| **Quintiles of the dietary contribution of ultra-processed foods (% of total dietary energy)^a^** | **BMI (kg/m^2^)** | | | **WC (cm)** | | | **Obesity (BMI≥30kg/m^2^)** | | | **Abdominal obesity^c^** | | |
|  | Mean | Mean difference^b^ | (95% CI) | Mean | Mean difference^b^ | (95% CI) | % | OR^b^ | (95% CI) | % | OR^b^ | (95% CI) |
| Q1 (lowest) | 25.7 | 0.00 | Ref. | 88.9 | 0.00 | Ref. | 15.6 | 1.00 | Ref. | 25.4 | 1.00 | Ref. |
| Q2 | 26.2 | 0.30 | (-0.17; 0.78) | 89.2 | 0.40 | (-0.24; 2.41) | 16.6 | 0.99 | (0.72; 1.37) | 30.8 | 1.24 | (0.95; 1.63) |
| Q3 | 26.3 | 0.51 | (0.07; 1.01) | 90.1 | 1.08 | (-0.25; 2.41) | 19.9 | 1.27 | (0.93; 1.74) | 28.1 | 1.11 | (0.84; 1.45) |
| Q4 | 26.9 | 0.97 | (0.44; 1.51) | 91.4 | 1.89 | (0.57; 3.22) | 21.7 | 1.34 | (0.98; 1.83) | 33.5 | 1.46 | (1.12; 1.90) |
| Q5 (highest) | 27.0 | 1.11^*^ | (0.51; 1.71) | 91.2 | 2.42^*^ | (0.87; 3.96) | 25.7 | 1.68^*^ | (1.21; 2.33) | 34.9 | 1.68^*^ | (1.27; 2.23) |
| Total | 26.4 | - | - | 90.2 | - | - | 19.9 | - | - | 30.6 | - | - |
| BMI: body mass index; WC: waist circumference; OR: odds ratio; CI: confidence interval | | | | | | | | | | |  |  |
| Ref.: Reference group | | |  |  |  |  |  |  |  |  |  |  |
| ^†^Excluding people following special diets or who reported diagnosis of diabetes, heart disease or kidney disease. | | | | | | | | | | | | |
| ^a^Percentage of energy intake from ultra-processed foods. Mean (range): All = 39.9 (0 to 100); Q1= 11.6 (0 to 19.9); Q2= 26.3 (19.9 to 32.2); Q3= 37.8 (32.2 to 43.5); Q4= 50.9 (43.5 to 59.8); Q5= 73.2 (59.8 to 100). | | | | | | | | | | | | |
| ^b^Adjusted for sex, age, educational attainment, income, zones, country of birth, level of physical activity and smoking status | | | | | | | |  |  |  |  |  |
| ^c^Defined as waist circumference ≥ 88 cm for women and ≥102 cm for men.  ^*^P-trend<0.001 | | | | |  |  |  |  |  |  |  |  |
